# Supplementary material for: High-entropy engineering of the crystal and electronic structures in a Dirac material
Source: Nat Commun. 2024 Apr 26;15:3532. doi: 10.1038/s41467-024-47781-9 (PMC11053097; doi:10.1038/s41467-024-47781-9)
Supplement: Supplementary file 1 — Supplementary Information [file 41467_2024_47781_MOESM1_ESM.pdf]

# —SUPPLEMENTARY INFORMATION—

# High-Entropy Engineering of the Crystal and Electronic Structures in a Dirac Material

Antu Laha,[1,*] Suguru Yoshida,[1,2,†] Francisco Marques dos Santos Vieira,[3] Hemian Yi,[1] Seng Huat Lee,[1,2] Sai Venkata Gayathri Ayyagari,[3] Yingdong Guan,[1] Lujin Min,[1,3] Jose Gonzalez Jimenez,[4] Leixin Miao,[3] David Graf,[5] Saugata Sarker,[3] Weiwei Xie,[4] Nasim Alem,[3] Venkatraman Gopalan,[3] Cui-Zu Chang,[1] Ismaila Dabo,[3,‡] and Zhiqiang Mao[1,2,3,§]

[1]*Department of Physics, Pennsylvania State University, University Park, PA 16802, USA*

[2]*2D Crystal Consortium, Materials Research Institute, Pennsylvania State University, University Park, PA 16802, USA*

[3]*Department of Materials Science and Engineering, Pennsylvania State University, University Park, PA 16802, USA*

[4]*Department of Chemistry, Michigan State University, East Lansing, MI, 48824, USA*

[5]*National High Magnetic Field Laboratory, Tallahassee, FL 32310, USA*

## Supplementary Note 1: Transport Property

### mobility ratio

We have estimated the mobility ratio between those from Fermi pockets at Γ point ($\mu^{\Gamma}$) and X point ($\mu^{\mathrm{X}}$), $\mu^{\Gamma}/\mu^{\mathrm{X}}$, as follows. According to the standard Drude formalism, mobility can be expressed in terms of electron charge ($e$), mean-free time ($\tau$), and effective mass ($m^*$):

$$\mu = \frac{e\tau}{m^*}. \qquad \text{(Supplementary Eq. 1)}$$

In addition, by using the relation of $m^*$ to Fermi velocity ($v_{\mathrm{F}}$) and Fermi vector ($k_{\mathrm{F}}$), i.e., $m^* = \frac{\hbar k_{\mathrm{F}}}{v_{\mathrm{F}}}$, one can obtain the following equation,

$$\mu = \frac{e\tau}{\hbar}\frac{v_{\mathrm{F}}}{k_{\mathrm{F}}}, \qquad \text{(Supplementary Eq. 2)}$$

where $\hbar$ is reduced Planck constant. Assuming that the mean-free time is the same for both the bands at Γ and X points, the relative mobility ratio can be written as

$$\frac{\mu^{\Gamma}}{\mu^{\mathrm{X}}} = \frac{v_{\mathrm{F}}^{\Gamma}/v_{\mathrm{F}}^{\mathrm{X}}}{k_{\mathrm{F}}^{\Gamma}/k_{\mathrm{F}}^{\mathrm{X}}}. \qquad \text{(Supplementary Eq. 3)}$$

The ARPES data shown in Supplementary Fig. 1 allows us to estimate $v_{\mathrm{F}}^{\Gamma}/v_{\mathrm{F}}^{\mathrm{X}}$ and $k_{\mathrm{F}}^{\Gamma}/k_{\mathrm{F}}^{\mathrm{X}}$ ratios based on the slope of the band dispersions and pocket sizes, respectively:

$$v_{\mathrm{F}}^{\Gamma}/v_{\mathrm{F}}^{\mathrm{X}} = 7.09 \times 10^{-2}, \qquad \text{(Supplementary Eq. 4)}$$

and

$$k_{\mathrm{F}}^{\Gamma}/k_{\mathrm{F}}^{\mathrm{X}} = 3.84. \qquad \text{(Supplementary Eq. 5)}$$

Finally, $\mu^{\Gamma}/\mu^{\mathrm{X}}$ is 1.8%, showing that the mobility of the band at Γ point is two orders of magnitude smaller than that at X point.

Additionally, we note that the band at the Γ point has some contributions from the orbitals of the $A$ cation, as seen from the atom-resolved band structures calculated for $BaMnSb_2$, $SrMnSb_2$, and $CaMnSb_2$ (Supplementary Fig. 2). This implies that in the high-entropy crystal, the transport originating from the Γ band is disturbed by the randomness at the $A$ site. Therefore, we expect that the $\tau^{\Gamma}$ is much smaller than $\tau^{\mathrm{X}}$, indicating that the actual mobility ratio is smaller than the value estimated above.

### Hall resistivity

The reason behind the statement "temperature-independent linear $\rho_{xy}$–$B$ curves indicate that the transport of this crystal is dominated by the linear Dirac bands" is the following. Within an

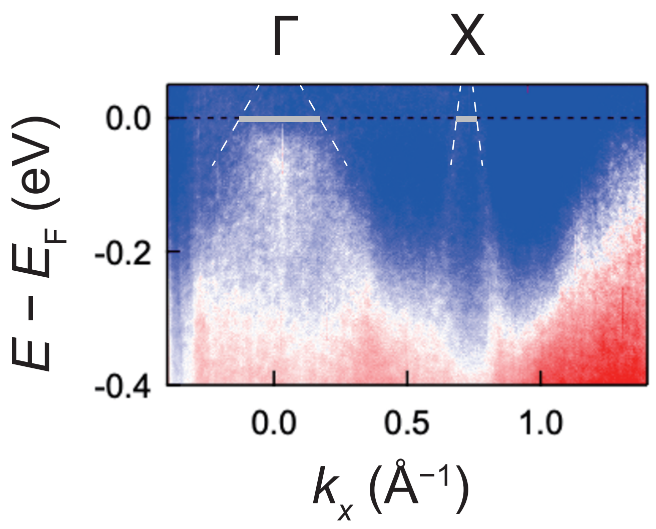


Supplementary Fig. 1: ARPES band mapping along the Γ–X direction (identical to Fig. 4(b) of the main manuscript) with the solid/dashed lines serving as the eye guide to show the pocket sizes and band slopes at Γ and X points.

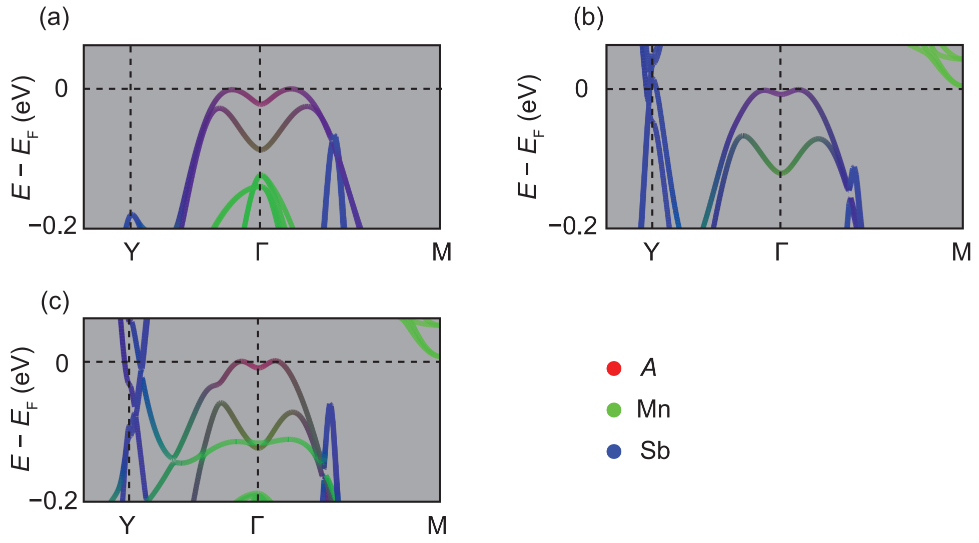


Supplementary Fig. 2: Atom-resolved band structure calculated for (a) $BaMnSb_2$, (b) $SrMnSb_2$, and (c) $CaMnSb_2$.

approximation of a single-band model, $\rho_{xy}$ depends linearly on the magnetic field $B$, whereas it deviates from linearity in the case of a two-band model. Thus, a linear $\rho_{xy}$–$B$ curve obtained for the high-entropy crystal suggests that either the hole pocket at the Γ or X point dominates the transport, not both the two. Given the very low mobility of the band at the Γ point compared with that at the X point as shown above, one can conclude that the transport behavior of this

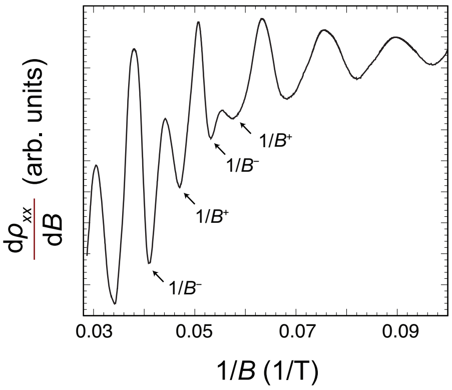


Supplementary Fig. 3: The first derivative of $\rho_{xx}$ (1.7 K and $\theta = 0°$) as a function of $1/B$.

compound is dominated by the band at the X point, i.e., the Dirac band. In addition, the slope of the $\rho_{xy}$–$B$, in principle, depends on the carrier density and thus temperature. However, the change in carrier density is expected to be small within a narrow temperature window (such as 2–50 K used for our measurements), and the $\rho_{xy}$–$B$ curve becomes almost temperature independent as shown in revised Fig. 5(a). Such behavior has also been confirmed in the pristine $BaMnSb_2$, for which the X-point hole pocket is proved to dictate the transport property via the observation and analysis of the quantum Hall effect[1]. Overall, we have concluded that linear $\rho_{xy}$–$B$ curves are indicative of the single-band (those at X point in this case) dominated transport of this crystal.

## Zeeman splitting

As mentioned in the main text, the Zeeman splitting of the quantum oscillation peaks is prominent at low temperatures (lower than 30 K). For clarity, we plot the first derivative of $\rho_{xx}$ as a function of $1/B$ in Supplementary Figure 3. For a particular Landau level, the spin-up and spin-down sub-Landau levels cross the Fermi energy at the magnetic field of $B^-$ and $B^+$, respectively. Using the following equation,

$$F_\alpha\left(\frac{1}{B^+} - \frac{1}{B^-}\right) = \frac{gm^*}{2m^0}, \qquad \text{(Supplementary Eq. 6)}$$

where $F_\alpha$, $m^*$, $m^0$, $g$ is oscillation frequency, effective mass, static electron mass, and Lande $g$-factor, respectively[2], we obtained 1.55 for $g$. This value is small compared to other Dirac semimetals such as $ZrTe_5$[3] and $Cd_3As_2$[4], but similar to the estimated values for pristine BaMnSb2 ($\simeq$2)[1].

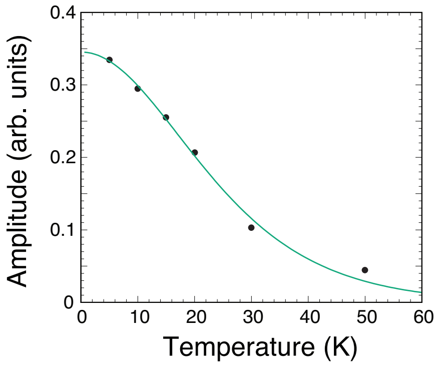


Supplementary Fig. 4: Temperature dependence of the fast Fourier transform (FFT) amplitude derived from $\frac{d^2\rho_{xx}}{dB^2}$ [Fig. 5(b)]. The fit of the Lifshitz-Kosevish (LK) formula is depicted by the solid line.

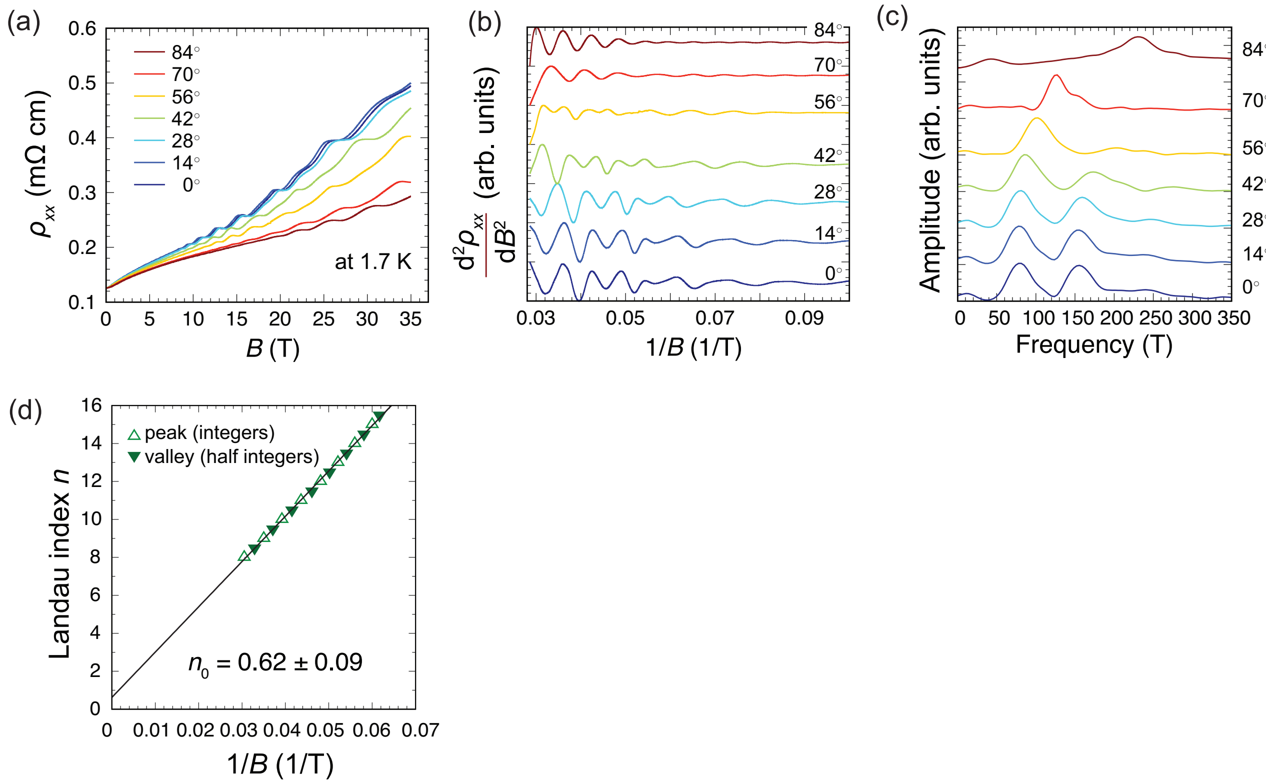


Supplementary Fig. 5: Additional magnetotransport characterizations for $A^5$MnSb$_2$. (a) In-plane resistivity $\rho_{xx}$ as a function of magnetic field $B$ at various angles $\theta$ (0°, 14°, 28°, 42°, 56°, 70°, and 84°), which is defined in the schematic shown in the inset of Fig. 5(d). (b) The second derivative of $\rho_{xx}$ at various angles as a function of $1/B$. (c) Angular dependence of the FFT spectra of $\frac{d^2\rho_{xx}}{dB^2}$. (d) Landau fan diagram obtained from $\frac{d^2\rho_{xx}}{dB^2}$ (at 1.7 K, $\theta = 84°$), where integers are assigned to the maxima of the second-derivative curve.

## Supplementary Note 2: Cyclotron Mass

The crystal structure (which depends on the size of $A$ cation) and the magnitude of spin-orbital coupling (i.e., the chemistry of the $X$ anion) dictate the band dispersion of $AMnSb_2$ at around the X point[5]. The structures without $X$-displacement ($P4/nmm$ and $I4/mmm$ structures, Fig. 1 of the main manuscript) exhibit massless Dirac crossing at the X point with spin-orbital coupling (SOC) excluded. On the other hand, activating SOC or $X$-displacement (zig-zag chain formation in $X_4$ layers) leads to a small gap at the Dirac point. Indeed, large electron masses have been observed for $YbMnSb_2$[6] ($0.134m_0$) and $SrMnBi_2$[7] ($0.29m_0$). Since the high-entropy compound also involves Sb-displacements as visualized by STEM analysis, we anticipated massive Dirac fermion behavior for this material as well.

The difference in the chemical potential between the bulk and surface, which is proved by transport and ARPES, respectively, would be the possible reason of the large cyclotron mass despite the linear dispersion seen in ARPES. Since the Dirac node is gapped, a slight deviation in chemical potential toward a less hole-doped regime pushes the Fermi energy to a nonlinear $E$–$k$ dispersion region, giving rise to a massive nature of the electron.

## Supplementary Note 3: Raman Spectroscopy

We measured the Raman spectrum to address the phonon behavior of the high-entropy crystal and compared it with those collected for parent compounds, $BaMnSb_2$, having the same point group $mm2$ as the high-entropy compound. Supplementary Figure 6(a) shows the Raman spectra at room temperature, where one can find that a peak of the high-entropy crystal is slightly shifted toward the low wavenumber region when compared to the $BaMnSb_2$. The ticks at the bottom of Supplementary Fig. 6(a) indicate the DFT-calculated Raman peak positions for $BaMnSb_2$ (PHONOPY code was used[8]). The phonon frequency may be underestimated because of the harmonic approximation and 0-K nature of DFT. Suppose we assign the strongest Raman peak to the theoretical peak position at around 135 $cm^{-1}$, the peak corresponds to an $A_1$ mode, for which eigendisplacement pattern is shown in Supplementary Fig. 6(b). Sb displacements in the $Sb_4$ layer is the main component of this phonon mode. Therefore, the peak shift toward the low wavenumber region observed for the high-entropy crystal implies the softening of Sb-displacement motion, probably due to the enlarged cavity caused by smaller cation accommodation than Ba ions.

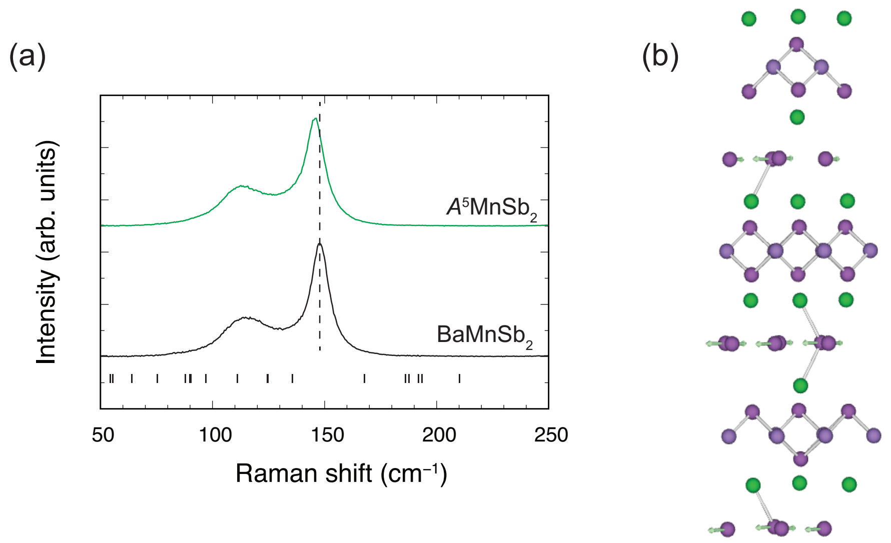


Supplementary Fig. 6: Raman spectroscopy. (a) Raman spectrum collected for $A^5MnSb_2$ and pristine $BaMnSb_2$. (b) Eigendisplacement of the $A_1$ mode of $BaMnSb_2$ ($\simeq$ 135 cm$^{-1}$).

### Supplementary Note 4: Second Harmonic Generation Measurement

We have measured SHG with the same optical setup for $YbMnSb_2$, a pristine compound with inversion symmetry, and the result is depicted in Supplementary Fig. 7(b) together with the data collected for the high-entropy crystal [panel (a), which is identical to Fig. 3(a) in the main text]. Please note that the scale of the radial axis of panel (b) is five times smaller than that of panel (a). Given the centrosymmetric nature, the surface-originated SHG is the main contribution to the signal from $YbMnSb_2$, but the intensity is within the background level. Because of the similarity of the structural motif among the two compounds as well as the fact that both the signals were collected from (001) plane, we expect that the surface contribution to the SHG signal in the high-entropy crystal is of the same order of magnitude. By comparing panels (a) and (b) of Supplementary Fig. 7, one can find that the signal from the high-entropy crystals is much stronger than that from $YbMnSb_2$ despite the comparable surface contributions. Therefore, we concluded that the SHG signal observed in the high-entropy crystal originates from the intrinsic effect due to the lifted inversion symmetry in the crystal structure with an almost negligible contribution from the surface.

### Supplementary Note 5: Comment on Stacking Fault

We acknowledge that it is very difficult to exclude the possibility of stacking faults and sample bending, which might give rise to extra reflection spots otherwise forbidded due to the crystal

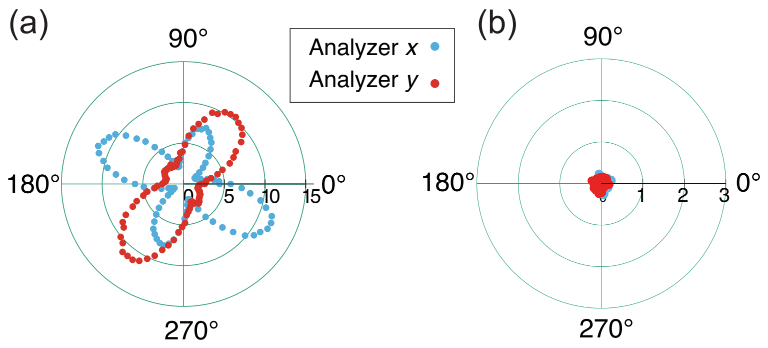


Supplementary Fig. 7: SHG comparison between $A^5$MnSb$_2$ and centrosymmetric YbMnSb$_2$. SHG intensity as a function of the polarization angle of the incident light collected for (a) $A^5$MnSb$_2$ and (B) YbMnSb$_2$.

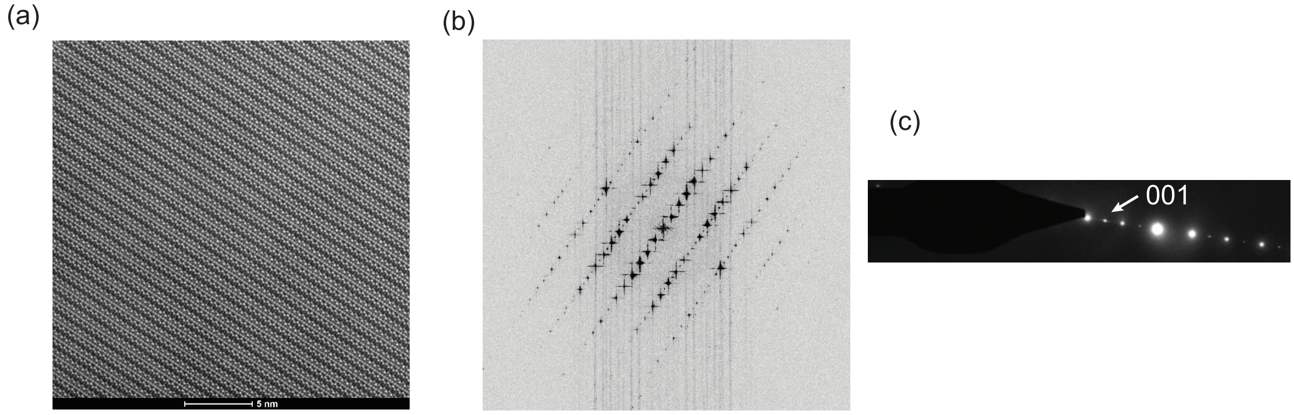


Supplementary Fig. 8: Additional STEM results obtained for $A^5$MnSb$_2$. (a) ADF-STEM image along [010] zone axis, (b) fast Fourier-transformed image of panel (a), and (c) enlarged view of SAED pattern [panel (d) of Fig. 3].

symmetry. For example, DiMasi *et al.*[9] studied LaTeSb with the space group of $Pmcn$ (a non-standard setting of $Pnma$), where the stacking of the Sb sheets is along the $c$ axis. They found forbidden $hk0$ ($h+k$: odd) reflections in the SAED patterns along [001] zone axis and attributed it to the stacking fault and/or sample bending. In our SAED experiment, $00l$ ($l$: odd) reflections were observed in the SAED pattern along [010] zone axis. Therefore, we would like to note that our situation is somewhat different from this reference.

We understand that it is important to make efforts to exclude the possibility of stacking fault and bending. Supplementary Figures 8(a) and 8(b) show the STEM image along [010] zone axis and its fast Fourier-transformed image, respectively. As seen in Supplementary Fig. 8(b), no streak line is observed, which suggests the absence of a stacking fault in this region. On the other hand, when the SAED pattern [Fig. 3(d) of the main manuscript] was thoroughly investigated, we did observe very weak streaks around the 001 reflection spot [Supplementary Fig. 8(c)]. Overall, we

cannot completely eliminate the possibility of stacking faults in this material. However, the streak is not continuous along $c^*$ direction, and thus we expect that the stacking fault is, if it exists, not so influential to allow otherwise forbidden reflections.

### Supplementary Note 6: Examples of the Structural Compromise

In addition to our example of $A^5$MnSb$_2$, there are a few cases where high entropy gives rise to different crystal or magnetic structures absent from the pristine systems. The former case is (Ti,Zr,Hf,Sn)O$_2$[10]. Although (Ti/Sn)O$_2$ and (Zr/Hf)O$_2$ crystallize in rutile and baddeleyite structures, respectively, the high-entropy phase adopts an $\alpha$-PbO$_2$ structure. The $\alpha$-PbO$_2$ structure is a higher-symmetry polymorph of baddeleyite, but the local coordination environment around the cation site resembles rutile (coordination number = 6) rather than that of baddeleyite (coordination number = 7). Again, the high-entropy structure can be regarded as a compromising result of distinct structures.

A similar compromising phase appears in the case of magnetic structure, as exemplified by (Gd,Tb,Dy,Ho)Mn$_6$Sn$_6$[11]. Gd and Dy favor the in-plane and out-of-plane spin ordering, respectively, whereas both Dy and Ho prefer tilted spin orientation (45° and 50°, respectively). As a result, the magnetic ground state of the high-entropy crystal has a 30° tilted spin orientation, which does not exist in any of the parent compounds.

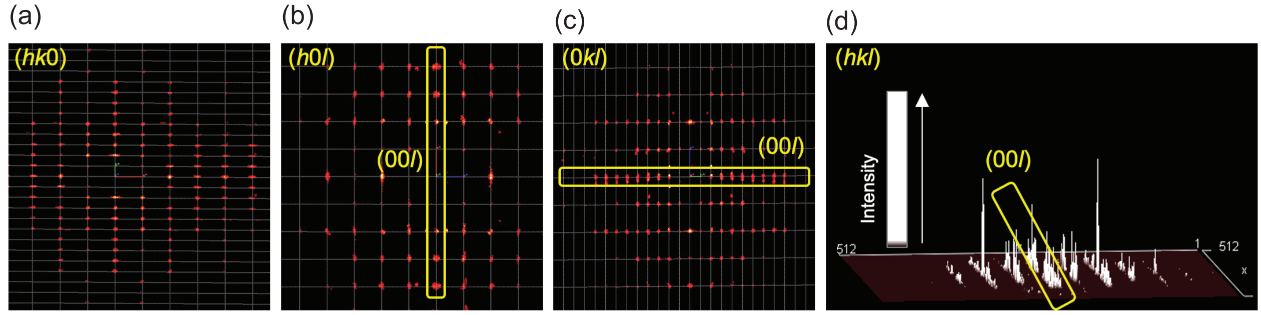


Supplementary Fig. 9: The Ewald sphere images generated from single crystal XRD at 300 K. 2D Images from (a) $(hk0)$, (b) $(h0l)$, and (c) $(0kl)$ and (d) 3D electron intensity map.

Supplementary Table 1: Single-crystal XRD refinements for $A^5MnSb_2$ with four structural models.

| Space group | $P2_12_12_1$ | $P2_1mn$ | $Pcm2_1$ | $Pc2_1n$ |
|---|---|---|---|---|
| $a$ (Å) | 4.3809(9) | 4.3809(9) | 4.3809(9) | 4.3809(9) |
| $b$ (Å) | 4.4143(9) | 4.4143(9) | 4.4143(9) | 4.4143(9) |
| $c$ (Å) | 22.779(5) | 22.779(5) | 22.779(5) | 22.779(5) |
| volume (Å$^3$) | 440.50(15) | 440.50(15) | 440.50(15) | 440.50(15) |
| Extinction coefficient | N/A | N/A | N/A | N/A |
| Flack factor | 0.29 | 0.73 | 0.76 | 0.69 |
| $\theta$ range (°) | 3.578–36.629 | 3.578–36.629 | 3.578–36.629 | 3.578–36.629 |
| # of reflections; $R_{\mathrm{int}}$ | 9174; 0.0723 | 9129; 0.0735 | 8650; 0.0751 | 8656; 0.0703 |
| # of independent reflections | 2002 | 2141 | 2199 | 1988 |
| # of parameters | 39 | 52 | 51 | 39 |
| $R_1$; $\omega R_2$ ($I > 2\delta(I)$) | 0.1168; 0.2359 | 0.1113; 0.2636 | 0.0984; 0.20777 | 0.1406; 0.3001 |
| $R_1$; $\omega R_2$ (all) | 0.1413; 0.2454 | 0.1392; 0.2760 | 0.1220; 0.2179 | 0.1644; 0.3094 |
| Goodness of fit | 1.0722 | 1.132 | 1.095 | 1.184 |
| Diffraction peak and hole ($e^-$/Å) | 10.397, −8.893 | 14.242, −17.484 | 10.9333, −8.308 | 9.013, −9.827 |
| R.M.S. deviation from mean | 1.151 | 1.243 | 1.172 | 1.224 |

Supplementary Table 2: Atomic coordinates and equivalent isotropic displacement parameters ($U_{eq}$) of $A^5MnSb_2$ obtained from refinement for single-crystal XRD data with a $P2_12_12_1$ model. ($U_{eq}$ is defined as one-third of the trace of the orthogonalized $U_{ij}$ tensor (Å$^2$)) The lattice parameters are listed in Supplementary Table 1.

| Atom | site | $x$ | $y$ | $z$ | Occupancy | $U_{eq}$ |
|---|---|---|---|---|---|---|
| Ba1 | 4*a* | 0.7564(7) | 0.0156(4) | 0.11295(9) | 0.933(1) | 0.018(1) |
| Sb1 | 4*a* | 0.2517(6) | 0.5169(5) | 0.17527(10) | 1 | 0.019(1) |
| Sb2 | 4*a* | 0.7531(7) | 0.5180(6) | 0.00073(11) | 1 | 0.024(1) |
| Mn1 | 4*a* | 0.2583(15) | 0.0198(12) | 0.2497(2) | 1 | 0.019(2) |

Supplementary Table 3: Atomic coordinates and equivalent isotropic displacement parameters ($U_{eq}$) of $A^5MnSb_2$ obtained from refinement for single-crystal XRD data with a $P2_1mn$ model. The lattice parameters are listed in Supplementary Table 1.

| Atom | site | $x$ | $y$ | $z$ | Occupancy | $U_{eq}$ |
|---|---|---|---|---|---|---|
| Ba1 | 2*a* | 0.7849(10) | 0 | 0.36273(13) | 0.97(1) | 0.013(1) |
| Ba2 | 2*a* | 0.2739(16) | 1/2 | 0.13679(17) | 0.87(2) | 0.030(1) |
| Sb1 | 2*a* | 0.2753(11) | 1/2 | 0.42502(13) | 1 | 0.012(1) |
| Sb2 | 2*a* | 0.7684(11) | 1/2 | 0.25031(17) | 1 | 0.017(1) |
| Sb3 | 2*a* | 0.7676(15) | 0 | 0.0745(2) | 1 | 0.040(1) |
| Sb4 | 2*a* | 0.2783(15) | 0 | 0.2490(2) | 1 | 0.043(1) |
| Mn1 | 2*a* | 0.783(3) | 1/2 | 0.5004(3) | 1 | 0.014(1) |
| Mn2 | 2*a* | 0.267(4) | 0 | 0.0003(5) | 1 | 0.046(1) |

Supplementary Table 4: Atomic coordinates and equivalent isotropic displacement parameters ($U_{eq}$) of $A^5MnSb_2$ obtained from refinement for single-crystal XRD data with a $Pmc2_1$ model. The lattice parameters are listed in Supplementary Table 1.

| Atom | site | $x$ | $y$ | $z$ | Occupancy | $U_{eq}$ |
|---|---|---|---|---|---|---|
| Ba1 | 2*b* | 1/2 | 0.5085(16) | 0.74188(19) | 0.84(2) | 0.025(1) |
| Ba2 | 2*a* | 0 | 0.99804(16) | 0.96787(16) | 1 | 0.016(1) |
| Sb1 | 2*b* | 1/2 | 0.0161(17) | 0.8533(4) | 1 | 0.043(1) |
| Sb2 | 2*a* | 0 | 0.9847(10) | 0.17973(19) | 1 | 0.021(1) |
| Sb3 | 2*b* | 1/2 | 0.4816(8) | 0.03031(19) | 1 | 0.018(1) |
| Sb4 | 2*a* | 0 | 0.4825(8) | 0.855(2) | 1 | 0.014(1) |
| Mn1 | 2*a* | 0 | 0.485(2) | 0.1051(6) | 1 | 0.020(2) |
| Mn2 | 2*b* | 1/2 | 0.979(2) | 0.1041(8) | 1 | 0.023(2) |

Supplementary Table 5: Atomic coordinates and equivalent isotropic displacement parameters ($U_{eq}$) of $A^5MnSb_2$ obtained from refinement for single-crystal XRD data with a $Pc2_1n$ model. ($U_{eq}$ is defined as one-third of the trace of the orthogonalized $U_{ij}$ tensor (Å$^2$)) The lattice parameters are listed in Supplementary Table 1.

| Atom | site | $x$ | $y$ | $z$ | Occupancy | $U_{eq}$ |
|---|---|---|---|---|---|---|
| Ba1 | 4$a$ | 0.2441(10) | 0.7442(9) | 0.11305(13) | 0.92(1) | 0.021(1) |
| Sb1 | 4$a$ | 0.22553(10) | 0.2360(16) | 0.00062(16) | 1 | 0.028(1) |
| Sb2 | 4$a$ | 0.7446(10) | 0.224(8) | 0.17523(14) | 1 | 0.022(1) |
| Mn1 | 4$a$ | 0.742(2) | 0.7331(3) | 0.2498(4) | 1 | 0.026(2) |

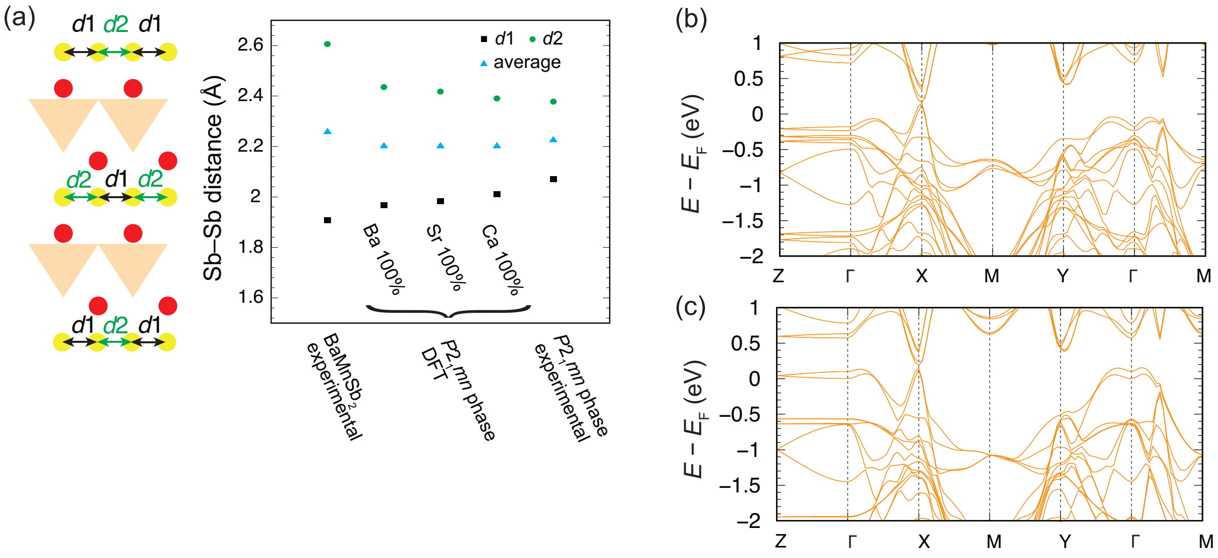


Supplementary Fig. 10: Additional DFT results. (a) Sb–Sb distances experimentally obtained for $BaMnSb_2$ ($I2mm$) and $A^5MnSb_2$ ($P2_1mn$) phases as well as those calculated for the $P2_1mn$ phase by DFT with assuming 100%-occupation of a single ion (Ba, Sr, or Ca) for the $A$ site. The distances $d1$ and $d2$ are defined in the schematic. DFT-calculated electronic band structure obtained by assuming (b) Ba 100% and (c) Sr 100% occupation for the $A$ site.

## Supplementary References


* These authors contributed equally to this work

† These authors contributed equally to this work; corresponding author:suguru.yoshida0224@gmail.com

‡ Corresponding author:dabo@psu.edu

§ Corresponding author:zim1@psu.edu



[1] J. Y. Liu, J. Yu, J. L. Ning, H. M. Yi, L. Miao, L. J. Min, Y. F. Zhao, W. Ning, K. A. Lopez, Y. L. Zhu, T. Pillsbury, Y. B. Zhang, Y. Wang, J. Hu, H. B. Cao, B. C. Chakoumakos, F. Balakirev, F. Weickert, M. Jaime, Y. Lai, K. Yang, J. W. Sun, N. Alem, V. Gopalan, C. Z. Chang, N. Samarth, C. X. Liu, R. D. McDonald, and Z. Q. Mao, Nat Commun **12**, 4062 (2021).

[2] A. Narayanan, M. D. Watson, S. F. Blake, N. Bruyant, L. Drigo, Y. L. Chen, D. Prabhakaran, B. Yan, C. Felser, T. Kong, P. C. Canfield, and A. I. Coldea, Phys. Rev. Lett. **114**, 117201 (2015).

[3] Y. Liu, X. Yuan, C. Zhang, Z. Jin, A. Narayan, C. Luo, Z. Chen, L. Yang, J. Zou, X. Wu, S. Sanvito, Z. Xia, L. Li, Z. Wang, and F. Xiu, Nat Commun **7**, 12516 (2016).

[4] J. Cao, S. Liang, C. Zhang, Y. Liu, J. Huang, Z. Jin, Z.-G. Chen, Z. Wang, Q. Wang, J. Zhao, S. Li, X. Dai, J. Zou, Z. Xia, L. Li, and F. Xiu, Nat Commun **6**, 7779 (2015).

[5] S. Klemenz, S. Lei, and L. M. Schoop, Annual Review of Materials Research **49**, 185 (2019).

[6] Y.-Y. Wang, S. Xu, L.-L. Sun, and T.-L. Xia, Phys. Rev. Mater. **2**, 021201 (2018).

[7] J. Park, G. Lee, F. Wolff-Fabris, Y. Y. Koh, M. J. Eom, Y. K. Kim, M. A. Farhan, Y. J. Jo, C. Kim, J. H. Shim, and J. S. Kim, Phys. Rev. Lett. **107**, 126402 (2011).

[8] A. Togo and I. Tanaka, Scripta Materialia **108**, 1 (2015).

[9] E. DiMasi, B. Foran, M. C. Aronson, and S. Lee, Phys. Rev. B **54**, 13587 (1996).

[10] S. S. Aamlid, G. H. J. Johnstone, S. Mugiraneza, M. Oudah, J. Rottler, and A. M. Hallas, Commun Mater **4**, 1 (2023).

[11] L. Min, M. Sretenovic, T. W. Heitmann, T. W. Valentine, R. Zu, V. Gopalan, C. M. Rost, X. Ke, and Z. Mao, Commun Phys **5**, 1 (2022).
